# A Survey of NFC Sensors Based on Energy Harvesting for IoT Applications

**DOI:** 10.3390/s18113746

**Published:** 2018-11-02

**Authors:** Antonio Lazaro, Ramon Villarino, David Girbau

**Affiliations:** Department of Electronic, Electric and Automatic Control Engineering, Universitat Rovira i Virgili, 43007 Tarragona, Spain; ramon.villarino@urv.cat (R.V.); david.girbau@urv.cat (D.G.)

**Keywords:** NFC, energy harvesting, IoT, green electronics

## Abstract

In this article, an overview of recent advances in the field of battery-less near-field communication (NFC) sensors is provided, along with a brief comparison of other short-range radio-frequency identification (RFID) technologies. After reviewing power transfer using NFC, recommendations are made for the practical design of NFC-based tags and NFC readers. A list of commercial NFC integrated circuits with energy-harvesting capabilities is also provided. Finally, a survey of the state of the art in NFC-based sensors is presented, which demonstrates that a wide range of sensors (both chemical and physical) can be used with this technology. Particular interest arose in wearable sensors and cold-chain traceability applications. The availability of low-cost devices and the incorporation of NFC readers into most current mobile phones make NFC technology key to the development of green Internet of Things (IoT) applications.

## 1. Introduction

Near-field communication (NFC) is a radio-frequency identification system (RFID) that enables fast communication between devices over a short range using the 13.56-MHz RFID band [1]. Although near-field communication has existed for over a decade [2], this technology did not become widespread until its extensive use in payment systems. NFC technology enables simple and safe two-way interactions between electronic devices, enabling consumers to perform contactless transactions, access digital content, and connect electronic devices with a single tap. Most current smartphones also incorporate an NFC reader. NFC systems are, therefore, gaining importance in the Internet of Things (IoT) scenario [3,4]. NFC is also interesting for the development of low-cost sensors since it provides a quick and easy way of obtaining data from them simply by approaching the reader to the tag without having to pair the devices. The upcoming fifth generation (5G) of communication technology is expected to unleash a massive IoT ecosystem where networks can serve the communication needs for billions of connected devices, with the right trade-offs between speed, latency, and cost. RFID is one of the most important technologies for the massive deployment of IoT. It can bring IoT to unpowered objects with its ability to connect the unconnected. In addition, NFC can put IoT devices under a user’s control and is easy to use with its “tap-and-go” nature. In particular, green NFC sensors based on energy harvesting can help in the design of a new generation of low-cost smart wearables and in the simplification of the man–machine interface, which opens the door to cooperative IoT for smart cities and Industry 4.0 applications.

Batteries in many electronic devices should be managed as hazardous waste because of their toxic contents or reactive properties [5]. In this context, green electronics technology provides solutions that are well suited to the broad needs of an energy-efficient society. Ambient energy harvesting is the process whereby energy is converted from the environment and stored for use in electronic applications [6]. This technology is usually applied to energy harvesting for low-power and small autonomous devices such as wireless sensor networks. Numerous sources, including solar power, ocean waves, piezoelectricity, thermoelectricity, and physical motion (active/passive human power), are available for energy scavenging [6]. Energy can be harvested from existing ambient radio-frequency sources (cellular networks, etc.). However, as available power is low and depends on distance and source availability [7], a long time is needed to charge the storage device (batteries or supercapacitors) before the device becomes operational. A review of the literature shows that no single power source is sufficient for all applications and that the energy sources must be selected according to the characteristics of the application [6]. From the energy-harvesting point of view, passive RFID systems can be considered devices that scavenge energy from an intentional RF source (the reader). Another technology related to energy harvesting is wireless power transfer (WPT) for battery charging using inductive links at high frequency (HF). Wireless battery charging was recently standardized by the Wireless Power Consortium (WPC) [8]. It became popular in modern smartphones [9] and was proposed for electric-vehicle charging [10].

In this context, interest in batteryless (passive) RFID sensors grew in the last few years. Ultra-high frequency (UHF) RFID technology is now commercially available and is used in logistics, inventory, and supply-chain applications. Although UHF readers are expensive ($1000–$2000), the inlays are cheap, and return on investment (ROI) is achieved thanks to a large number of units and the benefits associated with traceability [11]. Improvements in chip integrated circuit (IC) sensitivity (e.g., −22 dBm for Impinj Monza R6) allows reaching several meters (about 15 m considering free space and read mode). Consequently, the tag is more tolerant to environmental conditions such as losses or detuning due to the proximity of metals and materials with high permittivity and high losses, such as the body, or multipath fading [12]. In these cases, special designs to improve the read range are required [13,14]. The read range in write mode is noticeably lower than in read mode due to the higher power consumption needed during the memory writing process, and the write sensitivity is between 2 and 6 dB lower than in read mode, depending on the IC model. This not only permits a digital code to be associated with an object, but also allows RFID tags to be equipped with a large variety of sensors. Examples of UHF RFID sensors based on a modification of the antenna’s electromagnetic response [15,16] or integration of electronic pressure controller (EPC)-compatible integrated circuits with sensor functionalities [17,18] can be found in the literature. These can operate in passive mode or battery-assisted passive (BAP) mode. The EM4325 from EM Microelectronics integrates a temperature sensor and a serial parallel interface (SPI) connection to an external microcontroller, while the SL900 from Austria Microsystems integrates a temperature sensor and an analog-to-digital converter (ADC). The SPS1M002 from On Semiconductor uses the Magnus−S2 Sensor IC from RF Micron, which integrates a moisture contact sensor. In this case, changes in antenna detuning due to moisture contact are digitized by the sensor, which can then be read by a standard EPC Gen 2 compliant reader. Like Impinj R6, this IC technology incorporates an input internal tuning capacitance in order to combat the effect of detuning due to materials [19]. Several sensors (temperature, strain, and pressure) based on UHF technology were commercialized by Farsens using a proprietary IC. In passive mode, these UHF tags have read sensitivities on the order of −9 dBm (e.g., EM4325); therefore, the read range is considerably lower (about 2–3 m) than inlay tags without sensor capabilities. Another interesting technology is chipless RFID [20], where the identification code is encoded in the frequency or time domain. The read range is a few centimeters for the frequency-coded tags (<1 m) or up to 2–3 m for the time-domain-coded tags [21]. Several studies [20,22,23,24] focused on increasing the number of bits to encode in order to compete in cost with chip-based competitive technologies (HF or UHF). The number of bits depends on the frequency bandwidth; thus, chipless RFID is usually designed at ultra-wideband (UWB, 3.1–10.6 GHz). Another important challenge for chipless technology is the lack of standardized commercial low-cost readers. In spite of these challenges for identification, chipless technology aroused much interest in sensor applications [25,26,27]. Some challenges can be softened; for example, narrowband readers may be used since the identification can be done by other methods and the number of sensors in the read range may be small.

Concerning NFC, the most important NFC IC manufacturers, such as NXP, TI, ST Microelectronics, AMS, and Melexis, recently introduced advanced integrated circuits (IC) with energy-harvesting capabilities [28]. These chips collect part of the energy received by the magnetic field generated at the reader to provide an analog voltage output that can be used to power external electronics such as low-power microcontrollers or sensors. The progressive introduction of these ICs into the market enables the development of low-cost batteryless portable sensors [29,30].

A comparison of RFID technologies is shown in Table 1. Bluetooth low energy (BLE) is also included as an example of low-power, short-range wireless technology. The availability of low-cost standardized technology and the custom of users to use NFC technology and wireless power transfer makes NFC technology one of the keys to the development of a new generation of green sensors for IoT applications.

Recent advances in NFC-based sensor technologies are reviewed in this paper. The paper is organized as follows: in Section 2, several practical considerations for the design of NFC-based sensors are provided. Firstly, wireless power transfer between the NFC reader and the IC is described. After that, the factors that limit the read range such as antenna coupling, the quality factor of the antennas, and detuning due to the metallic surfaces are examined. In this section, a survey of existing NFC IC with energy-harvesting capability is also conducted. Several green sensors found in the literature are summarized in Section 3. Finally, some conclusions are drawn in Section 4.

## 2. NFC Energy Harvesting

**Figure 1:**
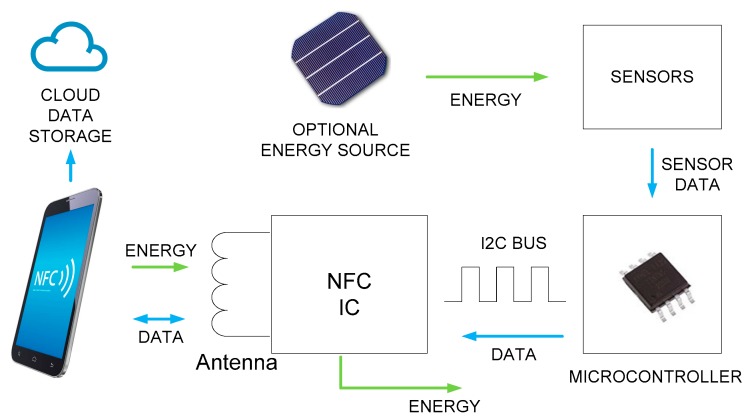
Block diagram of a green near-field communication (NFC)-based sensor system.

In batteryless mode, the tag is fully passive. In this mode, the NFC-enabled sensor harvests energy from incoming RF emissions (from a reader) to power the sensor interface and RF transmissions. In battery-assisted (semi-passive) mode, the NFC-enabled sensor can operate stand-alone in applications requiring autonomous and continuous monitoring working as data loggers. The life of a sensor tag may include operation in both modes: in semi-passive mode until the battery is exhausted, and thereafter, in passive mode. Data are stored in non-volatile memory and retained when the device is not powered. Figure 1 shows the block diagram of an NFC-based data logger assisted with a complementary energy source (e.g., solar cell).

**Figure 2:**
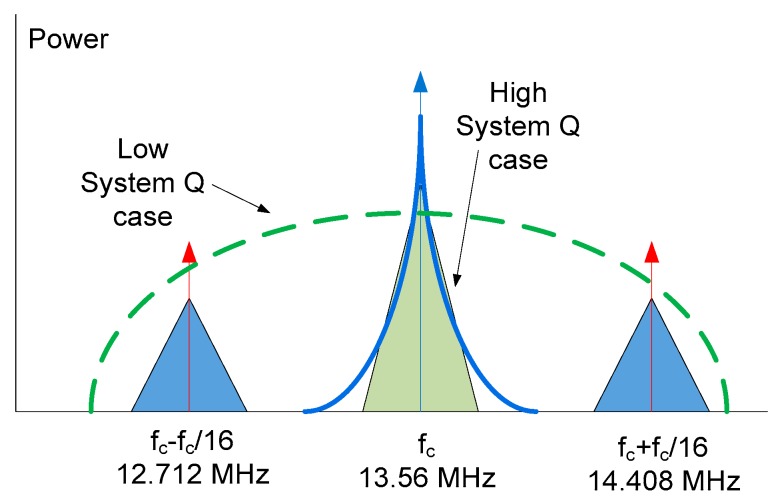
A typical spectrum of an NFC radio-frequency identification (RFID) system illustrating the reader command around the carrier frequency and the load modulation at the sidebands. The impact of increasing the reader *Q* factor is also shown.

NFC employs electromagnetic induction between two loop antennas. It operates within the globally available unlicensed radio-frequency industrial, scientific, and medical (ISM) band of 13.56 MHz on the ISO/IEC 18000-3 air interface at rates ranging from 106 to 424 kbit/s. Communication in NFC systems is based on inductive coupling between the reader and the tag antennas. The receiver antenna is connected to the internal tag rectifier, which takes energy from the RF field that is used to power up the tag electronics. The internal logic demodulates the amplitude shift keyed (ASK) message from the reader. The tag transponder (which is assumed to be passive) responds, using the passive load modulation technique, by changing the antenna impedance of the tag [1,31]. The passive load modulation spectrum consists of the RF carrier, two sidebands (at 12.712 MHz and 14.408 MHz), and modulated sidebands on these two subcarrier signals (Figure 2). All the transmitted data are carried in the two sidebands. The 13.56-MHz RF carrier, therefore, does not have to be transmitted by the transponder. The commands from the reader are transmitted in the sidebands of the carrier and the load modulation is carried in the sidebands of the two subcarriers shown in the blue triangles. Below, the conditions which must be met to enable communication between the reader and the tag are studied.

### 2.1. Link Budget

#### 2.1.1. Forward Link

**Figure 3:**
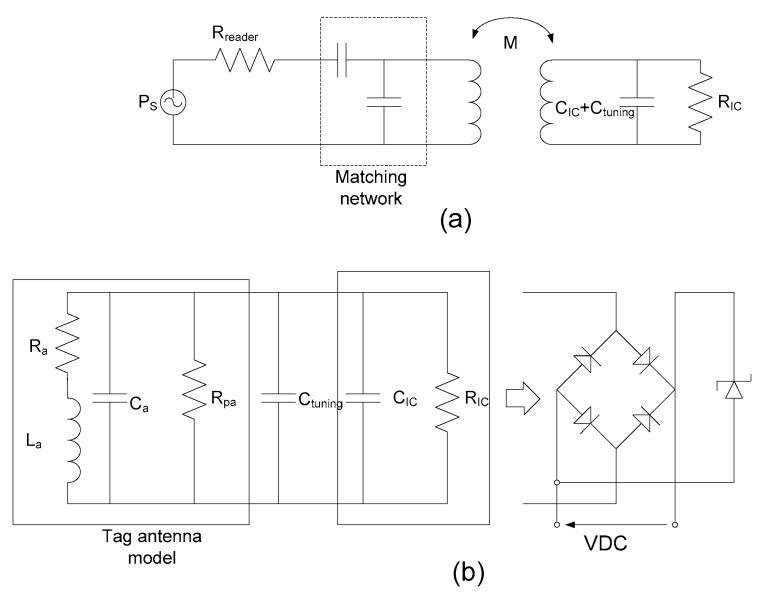
(**a**) Two-port transfer model between reader and tag; (**b**) tag model.

To establish communication between the reader and the tag (forward link) and to ensure enough power for the RF to direct current (DC) conversion to feed the electronic circuitry, the aim is to maximize power transfer from the reader to the tag. The power delivered to the tag IC (*P_d_*) must, therefore, be above a threshold power (*P_th_*):

(1) $$\;P_{d}=P_{s}G_{T}\left(f_{c}\right)>P_{th},\;$$

where *P_s_* is the power transmitted at the reader, and *G_T_* is the available gain at the center frequency, *f_c_*. To this end, efficiency must be maximized. The system is modeled as shown in Figure 3. Maximum efficiency is obtained from the available gain under matching conditions, *G_Tmax_*. This efficiency can be computed from the S-parameter measurements and expressed as a function of the parameter, *χ = k*^2^*Q*_1_*Q*_2_, where *k* is the magnetic coupling between the reader and tag coils ($k=M/\sqrt{L_{1}L_{2}}$) and *Q*_1_ and *Q*_2_ are the quality factors of the reader and tag coils, respectively [32].

(2) $$\;\frac{P_{dmax}}{P_{s}}=\eta_{max}=G_{Tmax}=\frac{\chi }{{\left(1+\sqrt{1+\chi }\right)}^{2}}.\;$$

**Figure 4:**
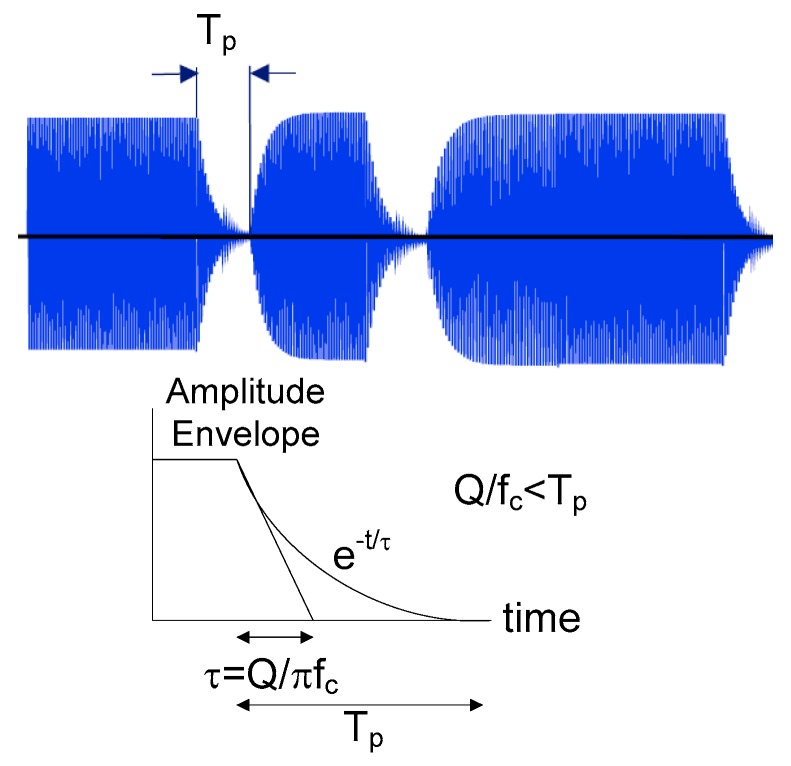
Typical time-domain modulated waveform (**top**) and idealized response of a second-order system with quality factor *Q* (**bottom**). The transitory response duration is of the order *f_c_*/*Q* < *T_p_*.

Power transfer can, therefore, be maximized by increasing the quality factor of the antennas or increasing the coupling that is a function of the distance between the antennas. However, a high *Q* factor would lead to limited bandwidth (see Figure 2) and long-time constants, causing severe distortion in the modulated signal (see Figure 4).

To ensure that the communication works properly, the maximum value of the *Q* factor of the initiator antenna must be such that the bandwidth B (at −3 dB), which is equal to *f_c_*/*Q*, is at least capable of channeling all the frequencies contained in the spectrum of the signal modulating the carrier frequency. The bandwidth of the forward link is the bandwidth of the modulation sidebands of the carrier and is dependent on the modulation scheme used by the reader. In the worst-case scenario (corresponding to the maximum value, *Q*_1*max*_, of the initiator antenna circuit), the fundamental bit rate of the square-wave signals (with a cyclic ratio of 50% in the case of non-return-to-zero (NRZ) bit coding) of that digital data stream must, therefore, be at least equal to half of the bandwidth B of the tuned circuit. The result is that *Q*_1*max*_ is limited to *f_c_/*(2 *×* bit rate). Unfortunately, for NFC uplink ISO 18092 (or ISO 14443, type A), in order to ensure maximum energy transference, the carrier is modulated using ASK (with 100% of the modulation index) and modified Miller coding. In this modified Miller bit coding, a pause on the carrier frequency of duration, *T_p_*, is made (see the top of Figure 4). This pause, *T_p_*, is equivalent to the transmission of a frequency whose period is 2*T_p_*, which is an equivalent bit rate of (1/2*T_p_*). Another interpretation in the time domain can be made. Figure 4 shows the amplitude envelope that decreases exponentially with a time constant, *τ* = *Q*/π*f_c_*. Assuming that the envelope will vanish after a few time constants, the reader *Q* factor is limited by Equation (3) [31].

(3) $$\;Q_{1max}=f_{c}\;\times \;T_{p}.\;$$

According to NFC forum standard ISO 14443, the quality factor is limited to 40 (35 considering design tolerances) at 106 kbit/s bit-rate transfers [31]. For applications that use NFC IP2–ISO 21481 with the authorized use of ISO 15693 and NFC–V targets, whatever the bit rate, the shortest time present in the uplink communication protocol is a “pause” lasting *T_p_* = 9.44 μs. In this case, *Q*_1*max*_ = 128, which generally can be reduced to *Q*_1*max*_ usable = 100 assuming design tolerances. Generally, these values of *Q*_1*max*_ usable are not difficult to obtain and are easy to reduce using serial resistors.

#### 2.1.2. Reverse Link

The green line in Figure 2 shows the magnitude of the frequency response for a low *Q* reader, while the blue line shows the magnitude for a high *Q* reader. We can see that, as the *Q* factor increases, bandwidth decreases and attenuation increases at the subcarrier frequencies. The return signal, therefore, becomes smaller due to increased attenuation. In this case, the return signal power (*P_b_*) is smaller than the reader sensitivity (*S_min,reader_*) and the reader cannot decode the load modulation. This condition in the reverse link can be mathematically expressed as

(4) $$\;P_{bs}=P_{m}G_{T}\left(f_{sub}\right)>S_{min,reader},\;$$

where *G_T_*(*f_sub_*) is the system transducer gain at the subcarrier frequency, and *P_m_* is the modulating power (*P_m_* = *m*^2^/4*P_d_*), which depends on the modulating factor, *m*. Typically, reader sensitivity is 110 dB below the level of the transmitter carrier signal (*S_min__,reader_* = −110 dBc) [1].

The read range can be limited by the forward link (Equation (1)) or by the reverse link (Equation (4)). In both cases, transducer gain is a function of the coupling coefficient between the two antennas, *k*. The coupling depends on the design, shape, area, materials, and distance of the antenna. The reader design may be different for different mobile devices. Differences in the read range are, therefore, expected depending on the coupling of each reader. The loaded quality factor of the tag is also not constant because the input impedance of the IC is nonlinear. For short distances, the IC receives high power and decreases load resistance and quality factor. The quality factor of the tag antenna is, therefore, not adjusted with external resistors, because it depends on the distance, which also simplifies the tag.

### 2.2. Tag Antenna Design Considerations

In order not to decrease the voltage that reaches the IC, a matching network is not used in the tag (as it is in the reader) that will introduce a voltage divider. Several studies [32,33] demonstrated that, for example, introducing a series capacitance for matching increases the backscattering modulated level but reduces the energy that reaches the IC. The tag design, therefore, consists of the design of the antenna and the adjustment of the tag resonance frequency with no matching network. From a system perspective, the analog RF performance of a batteryless transponder can be considered using a simplified equivalent circuit (shown in Figure 3b), where the IC chip is modeled as a parallel connection of a resistance (*R_IC_*) and chip capacitances (*C_IC_*). The tag’s resonance frequency must be tuned to the central frequency of operation or slightly shifted to a higher frequency to avoid detuning caused by the presence of metallic materials or the reader’s own loop. The tag’s resonance frequency is approximately calculated using Equation (5).

(5) $$\;f_{r}=\frac{1}{2\pi }\sqrt{\frac{R_{IC}+R_{a}}{L_{a}\left(C_{IC}+C_{p}+C_{tuning}\right)R_{IC}}}\approx \frac{1}{2\pi \sqrt{L_{a}\left(C_{IC}+C_{p}+C_{tuning}\right)}},\;$$

where *L_a_* is the tag’s antenna inductance, *C_IC_* is the internal IC capacitance, *C_p_* is the layout parasitic capacitance (which includes the antenna capacitance and the parasitic capacitance due to the interconnections), and *C_tuning_* is the capacitor used to adjust the resonance frequency to the operation frequency *f_c_* (13.56 MHz).

The antenna inductance, *L_a_*, can be calculated from compact analytical formulas [34], from numerical methods [35], or using full-wave electromagnetic simulators.

The antenna losses are the result of conductor DC resistance, and the alternating current (AC) losses are due to the skin effect. Depending on the substrate material, additional (e.g., dielectric) losses may also be significant. Parasitic capacitance for planar loop coils is a function of the conductor area, the gap between turns, the dielectric substrate, and permittivity. This can generally be extracted from the antenna’s resonance frequency. The losses in the dielectric are modeled with a resistance in parallel with the capacitor, *R_pa_*. This resistance can be neglected in antennas printed on low-loss substrates or in the air.

**Figure 5:**
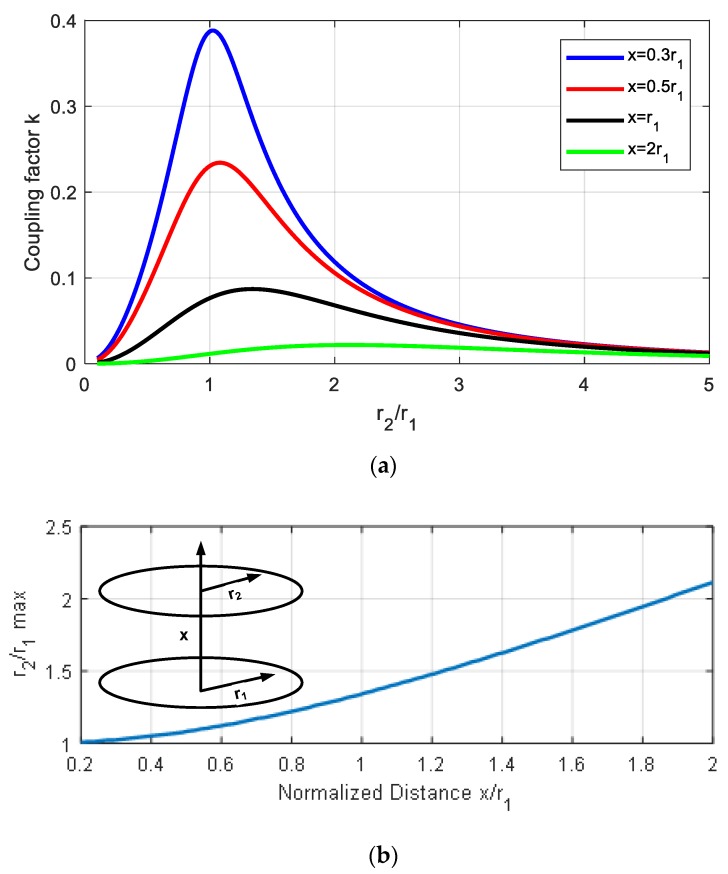
(**a**) Simulated coupling factor as a function of the radius of loop antennas *r*_2_/*r*_1_ for different axial distances. (**b**) Optimum ratio of the radius as a function of the normalized axial distance *x*/*r*_1_.

A key decision when designing the antenna is the size of the loop. Although this is restricted by the application, this decision plays a key role in the read range. To investigate the importance of the size of the antenna, the coupling between two circular loop antennas is considered as an example. Using Neumann’s formula, mutual inductance *M* can be found as a function of the complete elliptic integrals (e.g., implemented in MATLAB using the *ellipke* function). Analytical expressions can be derived for this case [36]. Figure 5a depicts the coupling factor *k* between two loop antennas of radius *r*_1_ and *r*_2_ as a function of the ratio between the two radii for different axis distances *x*. It can be derived that there is an optimum ratio *r*_2_/*r*_1_ that depends on the distance (see Figure 5b). The most widespread NFC readers are those integrated into smartphones. As the main application is for making payments, mobile antennas are often optimized to read payment cards (standard size = 85.60 × 53.98 mm). The reader radius *r*_1_ is, therefore, on the order of 2–2.5 cm. The typical read range is on the order of 1 cm. Figure 5 shows that the optimum case is when the two loop antennas have the same size (*r*_2_ ≈ *r*_1_). Although this conclusion is derived for the special case of circular loop antennas, the result can be extended to other shapes. 

**Figure 6:**
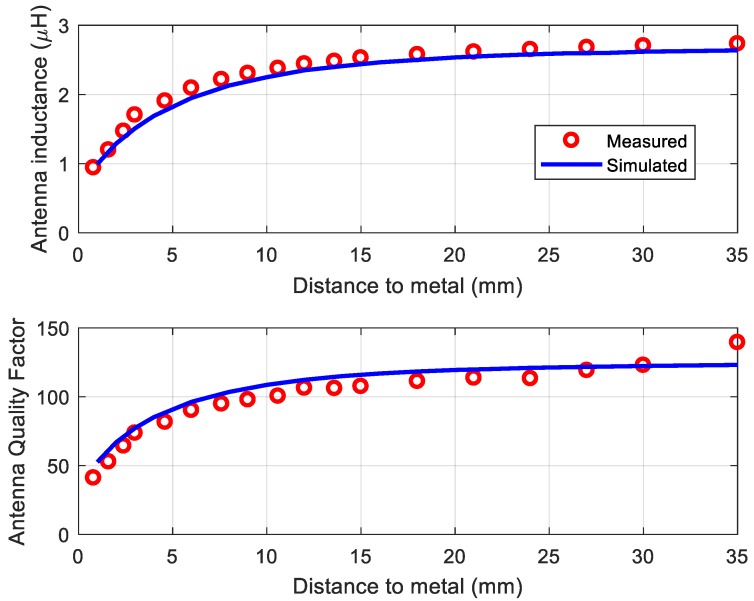
Simulated and measured antenna inductance (**top**) and quality factor (**bottom**) as a function of the distance to a ground plane [42].

Modern mobiles often use metallic cases; thus, a special design is used to avoid any losses introduced by the metallic parts or batteries [37,38,39]. Some mobiles use ferrites to avoid this problem [40,41]. Another factor that can reduce wireless power transfer is the detuning of the tag antenna when the mobile’s metallic parts are close to the tag. An example of a tag antenna was presented in order to study the effects of detuning [42]. Here, the antenna was designed using a two-dimensional (2D) full-wave simulator (Keysight-Momentum). To enable reading with a mobile NFC reader, a 50 × 50 mm loop antenna with six turns on a 0.8-mm-thick FR4 was chosen. The trace had a width of 0.7 mm and the gap between traces was 1 mm. By measuring the DC resistance, inductance, and resistance at the resonance frequency, the antenna model shown in Figure 3b could be obtained [42]. Figure 6 shows the inductance and quality factor of the tag antenna for several distances to a ground plane that simulates the mobile case. Agreement between the simulations and measurements is good. The measurements were taken by measuring the parameter *S*_11_ of a test antenna connected by means of a SubMiniature version A (SMA) connector to a vector network analyzer (VNA). The antenna impedance (*Z*) can be obtained from parameter *S*_11_ as a function of frequency for different antenna-to-metal distances. The antenna quality factor is obtained from *Q* = Im(*Z*)/Re(*Z*) at the operation frequency. An important reduction in inductance due to the induced image currents and an increase in losses due to the metal are observed. 

The resonance frequency of the tag can be adjusted using Equation (5) if antenna inductance, IC capacitance, and parasitic antenna capacitance are known. A practical procedure for adjusting the tuning capacitance (*C_tuning_* in Figure 3) can be conducted with a vector network analyzer. A test antenna (another prototype of the same antenna or a simple wire loop soldered to an SMA connector) is connected to port 1, and the S_11_ parameter is measured with the tag close to the mobile. The distance between the test antenna and the tag must be large enough (e.g., 1 cm) to avoid coupling between the antennas. After that, the tuning capacitance can be changed to tune the resonance frequency to the frequency of operation.

**Figure 7:**
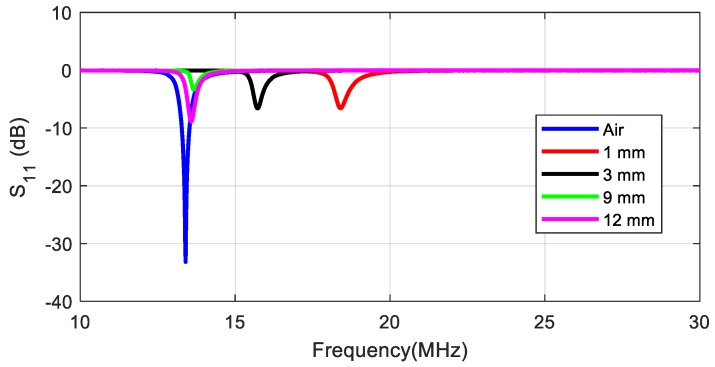
Measured S_11_ of the test antenna as a function of frequency for different distances between the tag and the mobile [42].

Figure 7 shows the *S*_11_ parameter measured for a tag adjusted in the air with *C_tuning_* = 22 pF and detuned due to the proximity of a mobile with a metallic case (model Huawei G8) at several distances between 1 and 12 mm. In order to solve this effect, the tag can be tuned again to 13.56 MHz for the desired distance by increasing tuning capacitance. Due to the power limitation of a standard VNA (often 20 dBm), the excitation field in this test is smaller than for a real reader. A modified VNA set-up with an external amplifier and a reflectometer was used in Reference [43] to characterize the tag under similar power conditions to the actual operation with a reader. IC impedance (especially the equivalent resistance) is, therefore, higher than for a real situation. The resistance of the chip typically decreases from *R_IC_* = 5 kΩ to 1 kΩ under high power excitation when the tag is very close to the reader [43]. The quality factor of the whole transponder *Q_T_* at the resonance frequency (which should not be confused with the antenna’s *Q* factor) can be derived from the parallel equivalent circuit of the antenna:

(6) $$\;Q_{T}=\frac{1}{\frac{R_{a}}{2\pi f_{r}L_{a}}+\frac{2\pi f_{r}L_{a}}{R_{IC}}}=\frac{R_{T}}{2\pi f_{r}L_{a}},\;$$

where *R_T_* is the tag’s total equivalent resistance, calculated by

(7) $$\;R_{T}=\frac{R_{IC}R_{pa}}{R_{IC}+R_{pa}}\approx R_{IC}.\;$$

The equivalent parallel resistance *R_pa_* can be derived from the series antenna resistance:

(8) $$\;R_{pa}=\frac{{\left(2\pi f_{r}L_{a}\right)}^{2}}{R_{a}}.\;$$

**Figure 8:**
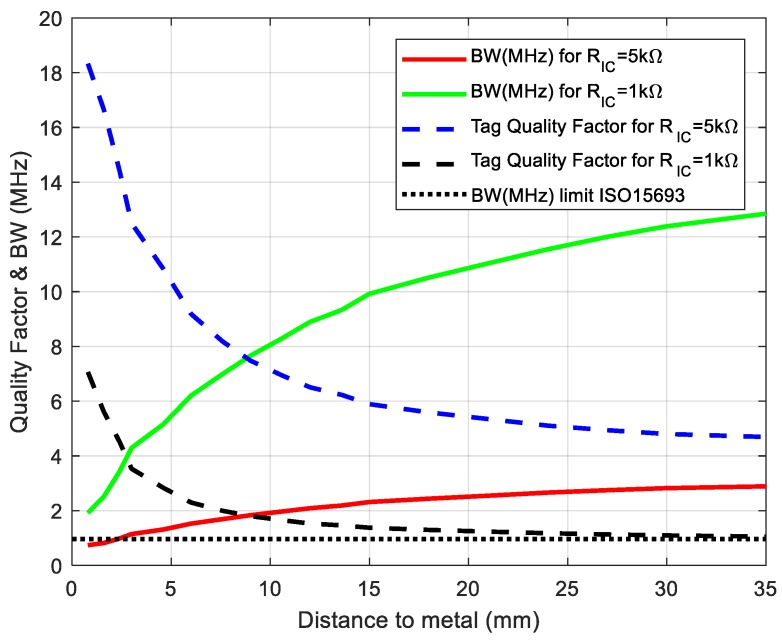
Tag quality factor and bandwidth (BW) as a function of the distance to metal depending on chip resistance *R_c_*. The BW limit for ISO 15693 is also shown.

The tag quality factor, therefore, decreases and the tag bandwidth (BW) increases with the increase in distance. When the reader–tag range decreases, the input voltage increases, and the rectifier gradually becomes conductive. On the other hand, the voltage-dependent parasitic capacitance increases by about 1.5% [43]. Therefore, the resonance tends to decrease due to the variation of the chip capacitance. Moreover, taking into account the presence of metal (smartphone case), the variation in the resonance frequency is masked since the inductance is significantly reduced due to the proximity to the metal. Consequently, an overall increase in the resonance frequency (see Figure 7) is produced. This result can be interpreted by analyzing Equation (5). However, because *R_pa_* >> *R_IC_*, the tag *Q* factor is mainly fixed by the chip resistance. Figure 8 shows the quality factor and the BW as a function of the tag-to-mobile distance. We can see that the BW is higher than that required for ISO15693 (968 kHz) and that shape distortion is due more to the reader than to the tag. 

### 2.3. Tag ICs with Energy Harvesting

Table 2 lists several NFC IC representatives with energy-harvesting capabilities. Some of these support ISO14443-3 or ISO15693. The table includes the maximum sink current (usable for external electronic devices such a microcontroller or sensors) and the nominal voltage (for low current consumption). The level of energy-harvesting voltage at the output is generated by the rectification of an RF signal in a non-regulated DC voltage that is only limited by the RF input clamping circuit. The maximum sink current is a function of the magnetic field present at the input. This value is obtained for the highest magnetic field; however, in most ICs, the voltage at this point decreases. Typically, currents around 5 mA for output voltages between 2 and 3 V and magnetic fields of the order of 3.5–5 A/m are obtained (e.g., 6 mA current and 1.7 V at 3.5 A/m is obtained for the M24LR-E-R or 4 mA and 3 V at 5 A/m for the ST25DV). Silicon Craft recently reported an IC with up to 10 mA (for the maximum field of 7.5 A/m) integrating an analog-to-digital converter (ADC) oriented to chemical sensors. Each series has a different memory size from 4 to 64 kbit and can be connected to other devices or microcontrollers using the I^2^C bus or serial-to-parallel interface (SPI). Although most of them are designed to be connected to a microcontroller, the MLX90129 from Melexis, the SL13 from AMS, and the SIC43x from Silicon Craft integrate an analog/digital (A/D) interface for autonomous sensor acquisition. Another special case is model RF430FRL152H from TI, which integrates a low-power microcontroller MSP430 and a 14-bit digital signal A/D interface.

For a well-designed batteryless tag, the main restriction in the read range is the power-up condition (Equation (1)) compared with the load modulation sideband amplitude (Equation (4)). Equation (1) can be expressed in terms of the magnetic field. For correct RF to DC conversion, the average magnetic field (*H_av_*) (Equation (9)) received by the NFC IC must be above a threshold H-field (*H_min_*). If the magnetic field is above that threshold, the harvesting voltage output can be below the desired value for the required current load. *H_av_* depends on both the reader and tag antennas and the coupling, and therefore, on the distance between the reader and the tag. *H_av_* is measured by the magnetic antenna factor, *AF* (Equation (10)). A procedure for calibrating the antenna factor of the tag antenna is described in Reference [44].

(9) $$\;H_{av}\left(A_{RMS}/m\right)=V_{RMS}\cdot AF,\;$$

(10) $$\;AF=\frac{Z_{0}+Z_{in}}{j\omega \mu_{0}Z_{0}A\cdot N},\;$$

where *A* is the loop area, *N* is the number of loops, *Z*_0_, is the reference impedance (50 Ω), and *Z_in_* is the input impedance of the antenna measured with the VNA. The root-mean-square voltage (*V_RMS_*) is obtained from the power *P* measured with a spectrum analyzer (*V_RMS_* = (*Z*_0_·*P*)^1/2^).

The minimum *H*-field as a function of tag resonance frequency can be described using Equation (11) [43].

(11) $$\;H_{min}\approx \frac{\sqrt{{\left[1-{\left(\frac{f}{f_{r}}\right)}^{2}\right]}^{2}+\frac{1}{Q_{T}^{2}}}}{2\pi f\mu_{0}A\cdot N}\cdot U_{min},\;$$

where *f_r_* is the resonance frequency of the tag, *Q_T_* is the total quality factor of the tag given by Equation (6), and *U_min_* is the minimum voltage required for the tag operation, which depends on the chip IC design and technology used. Equation (11) shows the importance of the tag resonance frequency being tuned to the operation frequency (13.56 MHz) and the highest tag quality factor for achieving a larger read range. In energy-harvesting tags, the maximum sink current for the sensor depends on the magnetic field. In order to increase this value, the tag is located at a lower distance compared to a conventional NFC tag, because an extra input power for the external devices is required. Therefore, the loading effect between tag and reader coils becomes significant when the distance decreases [43,45] resulting in detuning. Moreover, as shown earlier (e.g., Figure 6), the presence of metal under the tag can decrease antenna inductance and detune the tag, thus increasing *H_min_*. Other strategies for reducing *H_min_* involve increasing the tag antenna area or the number of turns. However, the increase in tag area forces an increase in reader antenna area in order not to degrade the coupling factor. In order to reduce the loading effect, it is important to choose the inductance value. Small inductance values, for instance, yield lower inductance voltages and require larger *Q*, while large inductance values require lower effective *Q* (larger number of coils, reduced current in the coil, and the resulting load effect is lower). However, from Equation (5), higher inductance results in smaller capacitances; therefore, the tolerances due to the layout fabrication and tuning capacitance must be reduced. In fact, less inductance (and more capacitance to result in equal resonance frequency) allows achieving higher chip currents, of course at the expense of increased loading and detuning of the reader [43].

Unfortunately, *H_min_* depends on parameters that are often not provided by the IC manufacturer, such as *U_min_*, harvesting power consumption, or other parameters that depend on the antenna and chip impedance, such as *Q_T_*. *H_min_* is independent of the reader used.

**Figure 9:**
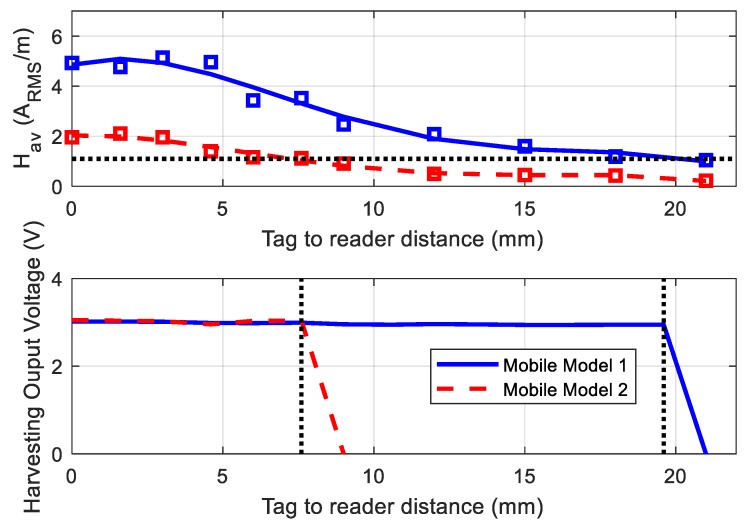
Measured average magnetic field (**top**) and harvesting output voltage (**bottom**) as a function of the tag-to-reader distance for two mobile models. We can see that, once the magnetic field goes below the threshold, the voltage output falls to zero.

The experiment carried out in Reference [42] showed how the minimum H-field can be obtained for a tag design with the target current consumption. Figure 9 compares the measured *H_av_* and the harvesting output voltage generated by two mobiles (Huawei G8 and Xiaomi Mi Note 2) as a function of the tag-to-mobile distance. Although mobile Model 1 generated higher power and the read range was wider, the threshold value was approximately the same *H_min_* = 1.1 A_RMS_/m. In this experiment, the NFC IC (M24LR04E-R) was loaded with the microcontroller (Attiny85 from AVR) and the sensors, in order to take into account the nominal current consumption (about 900 µA) under normal operation. It can be seen that the harvested output remained almost constant throughout the read range before the IC deactivated this output. 

If the application requires an NFC antenna to be very close to a metal plate or printed circuit board (PCB) electronics, a thin ferrite foil can help isolate the antenna from the metal [46,47,48]. In the case of the reader, it can also reduce interference from mobile circuits. Ferrite material can conduct the magnetic flux multiple times better than free air. The effect of the ferrite increases the antenna inductance by a factor *µ_ref_* (the definition of *µ_ref_* is analogous to the relative effective permittivity to take into account the increase of the capacitance on an inhomogeneous transmission line). The analysis performed earlier is valid if *µ*_0_ is replaced by *µ*_0_*µ_ref_* in Equation (11). The change in inductance leads to detuning of the tag in comparison with the case of air; therefore, tuning capacitance must be adjusted. Two types of ferrite foils are available on the market: polymer absorber sheets and sintered ferrite sheets. The former has higher losses, and the effective permittivity Re(*µ_r_)* is on the order of 20–60. The latter achieves higher Re(*µ_r_*), on the order of 100–190, and fewer Im(*µ_r_*) losses, typically 5–10 at 13.56 MHz (e.g., MHLL12060-000 from Laird). Ferrite foils can be ±15–20% the tolerance of *µ_r_*, which translates to tolerance in the antenna inductances. From Equation (11), after taking into account the correction in the effective magnetic permeability, we should expect *H_min_* at the resonance frequency to remain unaltered compared with the antenna in the air. However, the ferrite losses slightly reduce the total *Q* factor *Q_T_*, and *H_min_*(*f_r_*) with ferrite is slightly higher (roughly 15%) than in the case of air without ferrites [48]. Ferrite magnetic permeability is a function of temperature. Specific conductance has a significant temperature gradient. Inductance, quality factor, and resonance frequency are, therefore, temperature-dependent and *H_min_*, therefore, changes with temperature. This temperature dependence must be considered in industrial or automotive applications.

**Figure 10:**
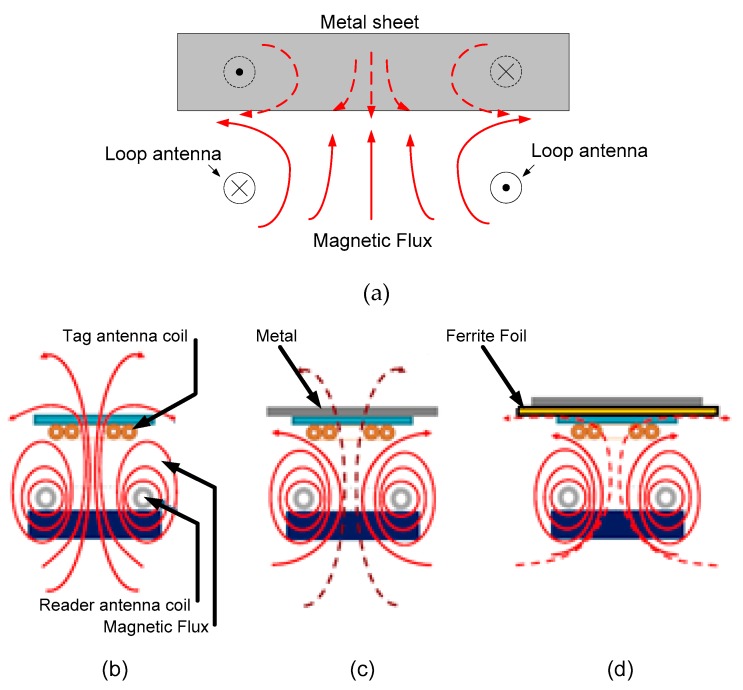
(**a**) Magnetic field on the surface of a metal sheet. Coupling between antennas (**b**) in free space, (**c**) with metal, and (**d**) with ferrite foil.

Since the direction of the magnetic field is almost parallel to the metal surface, a tag must be specially designed to obtain enough flux through the tag antenna coil surface. Figure 10a,c show that the magnetic field is parallel to the metal surface and that the magnetic flux is concentrated in the proximity of the coil [46], whereas the magnetic flux is zero in the center of the coil because of the cancelation of the field due to the image currents with the opposite sign. The field boundary conditions imposed by the ferrite make the magnetic field almost perpendicular, which is similar to the situation in the case of free space (Figure 10b,d). Reference [48] compared the operating distance between a sticker with ferrite foil composite and air coil in the presence of metal and reported that communication was not achievable with the air coil when the metal distance was less than 1 mm. On the other hand, a communication distance of 30 mm was obtained with a ferrite composite when the EMVCo test bench was used. Communication distance increased as the distance to the metal plate increased. When the metal distance was 10 mm, the air and ferrite composite reached the same communication distance.

### 2.4. NFC for Wearable Applications

One of the most interesting applications for NFC sensors is wearable applications for which the tag is on the body. With wearable devices, the effects of the body on the antenna must be taken into account. At this point, it is important to note that inductance is not affected by the dielectric substrate and is, therefore, unaltered by the body in applications where the tag is attached to the skin. However, parasitic capacitance (*C_p_*) increases due to the high permittivity of bodily matter. The tag’s resonance frequency must, therefore, take into account the body’s presence. The coupling coefficient is also essentially unaltered by the body’s presence. This panorama is dramatically different at UHF or microwave frequencies, where the high losses and the detuning of the antennas due to the body reduce the efficiency of the antennas, thus noticeably reducing the read range. Therefore, as we describe below, NFC technology is highly compatible with wearable applications. The dielectric losses can be modeled by adding a resistance in parallel to the antenna (*R_pa_* in Figure 3b). To quantify the effect of the body on the antenna, several simulations were performed. The same antenna as in Figure 6, printed on a 0.8-mm-high FR4 substrate was simulated in the air and on the body. It was assumed that the tag was on the arm, which was simulated using a planar stack of different dielectrics, as shown in Table 3. For the sake of simplicity, the curvature of the arm was ignored. The data for relative permittivity and conductivity were taken from Reference [49]. However, there was a large variation between individuals depending on the water content of their tissues. The results are shown in Table 4. Apart from the increase in capacity due to the high permittivity of the body, much deterioration in the quality factor was observed due to the losses. One solution is to isolate the body with a ferrite foil, as in the case of metal tags. A ferrite foil with a thickness of 100 µm (*µ_r_* = 120 − *j*5) and an adhesive layer of a thickness of 100 µm (*ε_r_* = 2.0, *tanδ* = 0.002) was inserted below the antenna. An increase in antenna inductance was observed due to the permeability of the ferrite foil and a smooth increase in losses compared to in the air. Another improvement when ferrite foil was inserted was that the design was insensitive to changes in individuals or parts of the body. One drawback, however, is that sintered ferrite sheets are expensive. Another simple solution to mitigate the effects of the body consists of introducing a spacer made with a low-permittivity material (such as a plastic *ε_r_* ≈ 2 or foam *ε_r_* ≈ 1) between the skin and the tag substrate. The separation of the antenna from the body significantly reduces the effects of the latter. In Table 4, simulated results for a spacer of foam (thickness: 1 mm) and plastic (thickness: 1 mm and 2 mm) are shown. In the case of plastic, to obtain results closer to the foam case (or air), it is necessary to double the thickness. The low-permittivity spacer allows for reducing the effective permittivity. Consequently, the parasitic capacitance decreases and the antenna resonance frequency increases, approaching the values expected for the air case. This solution is often implemented on wristbands [50,51] where the spacer is integrated into the belt, usually made with a biocompatible material such as silicone. In other cases, such as on body patch or tattoo tags, the thickness required is not allowed. The reduction of tag quality factor *Q_T_* due to the higher losses on the antenna caused by the presence of the body and the detuning due to the change of parasitic antenna capacitance increases the value of *H_min_*. In general, the first consequence is a reduction in the communication range; however, for NFC sensors with energy harvesting, the increase in *H_min_* reduces the maximum output current and output harvested voltage. Consequently, the sensor may not be powered up correctly. Thus, it is important to correctly adjust the tuning capacitance in the energy-harvesting NFC tags (see Table 4). Tags implemented with low inductance values (and adjusting the tuning capacitance for the resonance at the operation frequency) present lower sensitivity to variations in permittivity between persons or body parts. In addition, the use of a spacer helps further increase this tolerance. However, the area must be similar to the antenna used in the reader to maintain a coupling coefficient as high as possible.

### 2.5. NFC Reader Design Considerations

**Figure 11:**
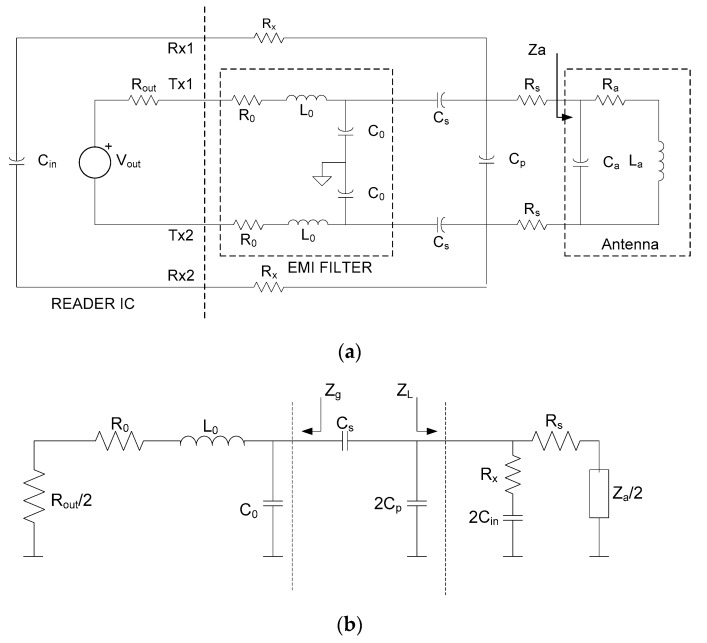
(**a**) Equivalent circuit of the reader, including the electromagnetic interference (EMI) filter and matching network. (**b**) Single-ended equivalent circuit.

Current mobiles recently incorporated NFC readers. NFC-based sensors are, therefore, normally read with these devices. However, certain applications (e.g., industrial ones) require a specific reader. Manufacturers of integrated circuits have solutions based on a low-cost single-chip reader. Such cases require the antenna for the reader and the control software of the microcontroller connected to the reader to be designed. To achieve maximum read distance, maximum power must be transmitted to the antenna; thus, the impedance of the transmitter must be the conjugate of the antenna. For this, a matching network must be designed. In addition to reducing electromagnetic (EM) interference with other systems, a low-pass band filter is inserted after the transmitter to attenuate the harmonics of the transmitted signal. This filter introduces some extra distortion into the signal and increases the bandwidth (or reduces the quality factor). A simple L-matching network is used as the matching network. In practice, the Tx output is usually differential (to enable double output voltage swing from a single supply voltage). Figure 11a shows a model of a differential reader with an EM interference (EMI) filter (capacitance *C*_0_, inductor *L*_0_, and inductor losses *R*_0_), the matching network, which consists only of capacitances (*C_s_* and *C_p_*), and the antenna model. The antenna is assumed to be an inductive load (the antenna resonance frequency is higher than the operation band). This load impedance often falls within the allowed region on the Smith chart that can be matched with an L-matching network with two capacitors. The output transmitter resistance *R_out_* depends on the transmitter’s current consumption and, therefore, on the transmitted power, and is generally given by the IC manufacturer. HF capacitors (with C0G or NP0 dielectric) have negligible losses and less tolerance. The input impedance from the receiver is typically capacitive and is modeled as capacitance *C_in_*. The resistance *R_x_* is inserted in order to attenuate the transmitter signal and to avoid receiver saturation. The antenna model can be obtained from electromagnetic simulations or from S_11_ measurements with a VNA [27]. The *C_a_* capacitance is derived from the antenna’s unload resonance frequency. The design procedure is described below.

1. Design of EMI filter: a filter cut-off is chosen between 15–20 MHz and is given by

(12) $$\;f_{cut}=\frac{1}{2\pi \sqrt{L_{0}C_{0}}}.\;$$

2. Adjustment of the maximum quality factor: the second step is to adjust the quality factor to make it equal to *Q*_1*max*_. In both this step and the design of the matching network, it is assumed for the design criteria that the tag is far enough away to make the coupling very weak. Tag proximity, therefore, has no influence on the load impedance and the tag is matched for small couplings, where efficient wireless power transfer is more important. If the tag is close to the antenna reader, the main effect is a reduction in the reader quality factor, which is given by [52]

*Q*_1_ = *Q*_1*u*_/(1 + *k*). (13)

This reduction in quality factor leads to an increase in matching bandwidth. The unloaded quality factor (*Q*_1*u*_) is adjusted by adding two series resistance *R_s_* with values of

(14) $$\;R_{s}=0.5\left(\frac{2\pi f_{c}L_{a}}{Q_{1max}}-R_{a}\right).\;$$

3. Design of the matching network: at this point, it is useful to design the L-matching network to use the single-ended equivalent circuit in Figure 11b. The values of *C_s_* and *C_p_* can be found with the help of the Smith chart or from the following equations:

(15) $$\;C_{p}=\frac{-B_{L}-\sqrt{\frac{G_{L}}{R_{g}}-G_{L}^{2}}}{2\omega },\;$$

(16) $$\;C_{s}=\frac{1}{\omega \left(X_{g}+Im\left(\frac{1}{Y_{L}+j\omega 2C_{p}}\right)\right)},\;$$

where *Y_L_* = 1/*Z_L_* = *G_L_* + *jB_L_* is the load admittance (see Figure 11b), and *Z_g_* = *R_g_* + *jX_g_* is the impedance at the input of the matching network (see Figure 11b).

4. Adjustment of the attenuation of the receiver path: the resistance *R_x_* controls the attenuation of the signal to the receiver. Usually, this resistance is high and the effect on the design of the matching networks is small. It is recommended that this adjustment should be made after checking with an oscilloscope with a low-capacitance probe that the voltage at the receiver input (*R_x_*_1_ or *R_x_*_2_) does not exceed the limit given by the reader manufacturer.

## 3. NFC Sensors

**Figure 12:**
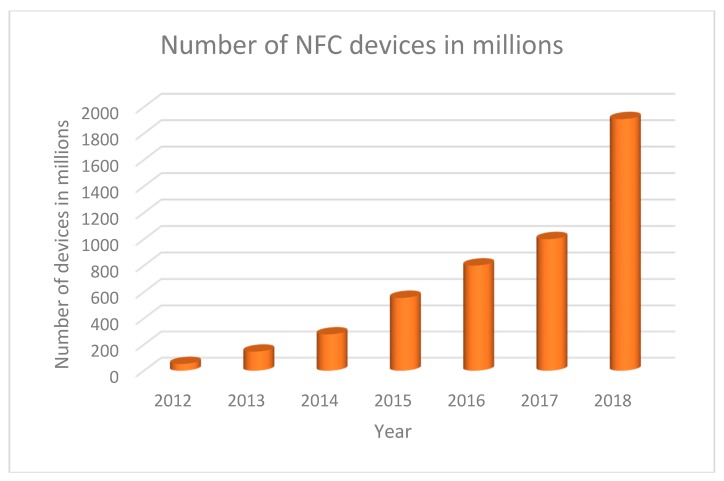
Number of NFC-enabled mobile devices worldwide from 2012 to 2018 (in millions of units).

The progressive introduction of NFC ICs into the market enables the development of low-cost batteryless portable sensors. Figure 12 shows the number of NFC-enabled mobile devices worldwide between 2012 and 2018 [53,54]. Between 2013 and the end of 2018, worldwide shipments of NFC-enabled cellphones rose by 325%. Market estimations expect that, by 2020, 85% of smartphones will be equipped with NFC. In this section, we review several NFC-based sensors in the literature. Some of these sensors are listed in Table 5. The second column in this table describes the target application or sensor type. The third column shows the NFC IC used in commercial devices or custom IC designs. Although we focused on passive devices, we also included some interesting semi-passive (battery-assisted passive tags) or data logger implementations. Several comments are also included. The references were sorted based on application.

One pioneering work is the NFC-WISP platform [29]. In this case, rectification is done externally using a full-wave rectifier with discrete diodes, and the ISO-14443 protocol is completely implemented in a low-power microcontroller (TI MSP430). Optionally, an E-ink screen can be used to show the measurements. In this case, a thin-film battery or supercapacitor must be used in order to provide the peak current for the E-ink screen. Temperature measurement for cold-chain data logging is shown as an RFID data logger, which provides temperature history for personnel without post-processing via the E-ink screen. Reference [55] reports a system inspired by the NFC-WISP design for monitoring the temperature of newborns in an incubator. The tag was positioned on the mattress inside the incubator and the reader (TI TRF7970A) was placed below the mattress tray. Temperature was recorded periodically.

Another implementation for cold-chain temperature monitoring and quality is found in Reference [56], which developed a critical temperature indicator (CTI) based on a solvent melting point. The smart sensor combines irreversible visual color changes and RFID. A Melexis MLX90129 was used to measure the change in resistance of multi-walled carbon nanotubes (MWCNTs) connected to two copper wires. The proposed CTI smart sensor integrates the microfluidic CTI to an RFID tag in order to remotely detect the melting of the solvent once the critical temperature is reached. The CTI smart sensor has a fast response to the critical temperature of 18–19 °C.

The medical market is especially poised to take advantage of NFC thanks to smart sensors that can measure the physical conditions of patients and wirelessly transmit the data to a nearby smartphone [57]. The measuring of vital signs for personalized healthcare is generating substantial interest from ambient assisted living solutions [58,59]. NFCs provide an intuitive user interface that is easy for patients to use. The latency between touching the device and displaying the result is typically less than one second. The main properties of these sensors are that they are wearable, low-cost, and green. Moreover, the tags can be disposed of in order to avoid contamination between patients. Smartphones enabled with NFC technology facilitate integration with cloud services because the same app that is used for sensor data reading can upload the data to the cloud using a mobile or WiFi internet connection. The continuous monitoring of medical parameters can help improve the diagnosis and follow-up of several diseases, while also reducing personal attention. In the long term, incorporating these sensors can help reduce the cost of healthcare in societies with aging populations. Another potential application is the development of devices for fast screening before deciding whether more expensive analysis is required.

One example of the above is the design of biopatches for body temperature monitoring (see Reference [60]). Here, the sensor was based on a thermistor and a Wheatstone bridge, where the internal ADC of the RF430FRL152H NFC IC from Texas Instruments was used. The biopatch can be used as a data logger if a small 1.5-V battery (with 30 days of autonomy) is used or in passive mode for instant temperature measurement. Analog inputs are used to read the temperature sensor, and the values read by the ADC are stored in the ferroelectric random-access memory (FRAM) to be downloaded when required. The timer is responsible for managing the time intervals. Another example of a biopatch for measuring temperature or light intensity, implemented in the form of an adhesive E-tattoo, was presented in Reference [61]. An RF430FRL152H NFC IC drives a light-emitting diode (LED) and a phototransistor that is able to detect backscattered or ambient light. Analog signals from sensors such as the thermistor and phototransistor are digitized with the ADC inside the NFC chip. These data are then transmitted by NFC. A Cu foil is laminated on thermal release tape (TRT). The circuit is made with a mechanical cutter plotter and is transferred onto water-soluble tape (WST) backed by Kapton tape. The NFC chip and the components are attached with solder paste. By dissolving the WST with water droplets, the whole circuit is transferred to the target substrate, which is water-vapor-permeable Tegaderm adhesive. Finally, another Tegaderm layer is used to provide protection from the skin.

A biopatch for continuously monitoring hydration was reported in Reference [62]. The sensor, which measures the concentration of NaCl in sweat, was based on Melexis MLX90129, which is used for the potentiometric sensing of electrolytes in sweat, reading surface temperature, and sensing the potential difference between two electrodes. The flexible printed circuit board (PCB) was built from Dupont Pyralux. Double-sided medical adhesive tape is used below the patch, while, above the patch, a medical textile covering is added to protect it and improve visual aesthetics.

A non-invasive, flexible, and wireless pH-sensing system for monitoring wound healing and identifying the possibility of early-stage infection was reported in Reference [63]. Low pH is beneficial since it helps counteract microbial colonization from many human-pathogenic microorganisms that require a more alkaline environment for growth. The sensors consist of a working electrode and a reference electrode. The electrical potential across the two electrodes is a function of the concentration of H^+^ ions in the solution. The sensor is interfaced to an NFC SL13 chip from AMS with a buffer amplifier (AD8603, Analog Devices Inc., Norwood, MA, USA). The pH sensor exhibits a linear sensitivity of −55 mV/pH and stable performance under mechanical bending in a pH range of 4 to 10. 

A low-power complementary metal-oxide semiconductor (CMOS) ion-sensitive field-effect transistor (ISFET) array for pH sensing was inductively powered using NFC in Reference [64]. Each pixel in a 3 × 3 array contains an ISFET operating in weak inversion that detects changes in pH as a current. The output for all pixels is then averaged, and the resulting signal modulates the frequency of a ring oscillator. This provides simple analog-to-digital conversion suitable for reading and transmitting. The application-specific integrated circuit (ASIC) power consumption was 6 µW (at a 1.2 V supply). The SIC4310 from Silicon Craft NFC IC was used in the study.

The literature contains specialized ASIC designs that integrate sensing, signal processing, energy harvesting, and NFC communication. A batteryless wearable electrocardiogram (ECG) monitoring system-in-a-patch assembled by biocompatible and pliable silicon-in-parylene technology was reported in Reference [65]. The system is able to process the acquired ECG signal and detect arrhythmia using a built-in digital signal processor (DSP). An NFC communication system is used to interface the external reader. The system requires an additional power source. The energy harvested from a 5 × 5 cm^2^ thermoelectric generator (TEG) module (60 W of output power) can be powered and stored in a supercapacitor.

An integrated system-on-chip (SoC) for long-term implantable continuous glucose monitoring was reported in Reference [66]. This integrates an amperometric glucose sensor interface, an NFC wireless front-end, and a fully digital switched mode power management unit for supply regulation and on-board battery charging. It uses the 13.56-MHz (ISM) band to harvest energy and backscatter data to an NFC reader. However, it does not use standardized protocol, and custom ASK demodulator circuits extract the modulating frequency that encodes the glucose concentration.

Another ASIC for a wireless fully implantable glucose sensor was reported in Reference [67]. In this case, the NFC was based on ISO15693 for passive wireless readout through an NFC interface. The IC is used as the core interface to a fluorescent, glucose transducer to enable a fully implantable sensor-based continuous glucose monitoring system. The whole system (photodiodes, transimpedance amplifier (TIA), ADC, electrically erasable programmable read-only memory (EEPROM), and NFC), except for an external LED, is integrated into the IC.

Chemical gas sensors based on NFC technology were recently reported in the literature [68,69]. Portable gas sensors are used for diagnosing point-of-care diseases, detecting explosives and dangerous chemical agents, indicating food ripening, and monitoring environmental pollution [69]. Reference [68] presents a fully passive flexible multigas-sensing tag for determining oxygen, carbon dioxide, ammonia, and relative humidity, readable by smartphone. The tag is based on NFC technology for energy harvesting and data transmission to a smartphone. The gas sensors show an optic response that is read through high-resolution digital color detectors. A white LED is used as the common optical excitation source for all sensors. The responses of the sensors were calibrated and fitted to simple functions, thus allowing fast prediction of gas concentration. Another gas sensor detection system was presented in Reference [69]. Sensitized single-walled carbon nanotubes (SWCNTs) whose resistance changes with gas concentration (NH_3_) were inserted in series with the NFC IC. The effect of the gas causes the tag to detune. When gas concentration is high, power transfer is insufficient for effective smartphone–tag communication and the tag is unreadable.

An NFC bicycle tire-pressure measurement system (BTPMS) was presented in Reference [70]. The sensor comprises an ASIC that integrates an on-chip capacitive pressure and temperature sensor, an RFID interface for HF/NFC, and EEPROM. The IC is soldered with wire-bonding to a FR4 PCB with the antenna. The tag is incorporated into the bicycle tire. A marker on the tire’s exterior indicates the position of the NFC BTPMS, and therefore, the NFC readable area. The pressure can be read using an ISO 14443 RFID-compatible reader. As the sensor presents linear dependence, a two-point calibration technique is sufficient for sensor calibration.

**Figure 13:**
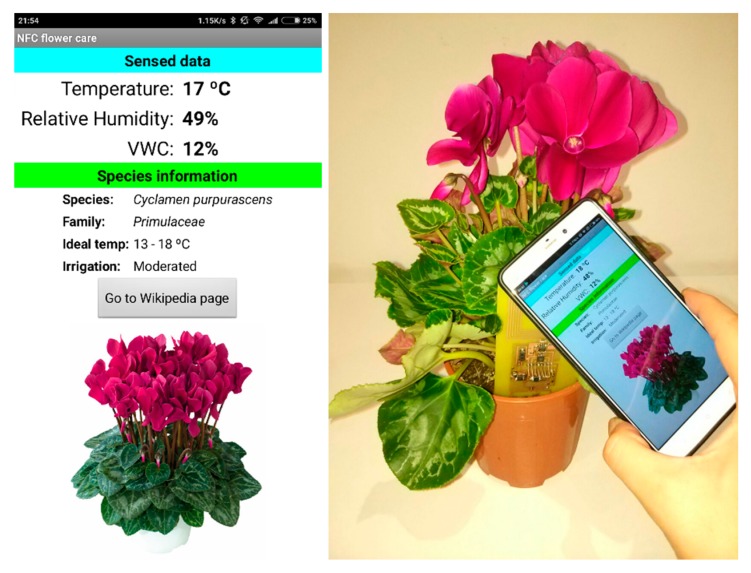
Soil moisture NFC tag being powered and read by a smartphone, which retrieves the sensed data and the tag unique identifier (UID) to identify the species [71].

**Figure 14:**
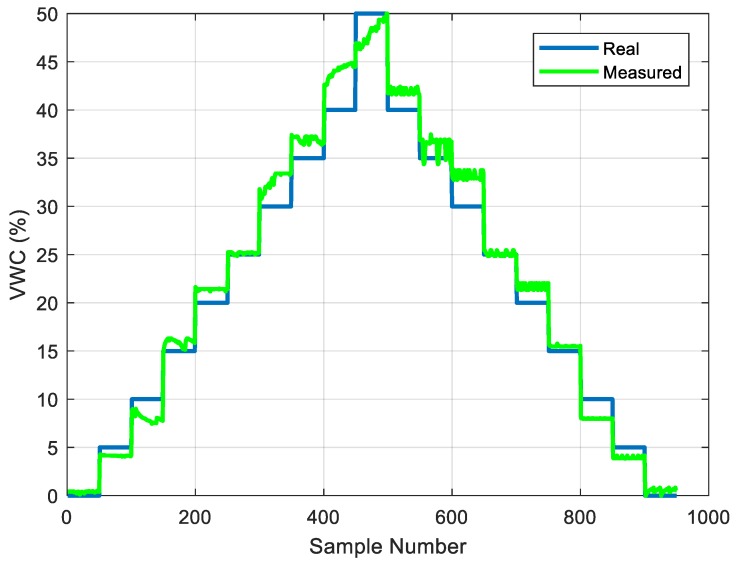
Comparison of real volumetric water content (VWC) and that obtained with the sensor [71].

Low-cost monitoring systems are in demand for irrigation control at home, in greenhouses, or at garden centers. A low-cost, batteryless, NFC-powered device capable of measuring volumetric water content (soil moisture), temperature, and relative humidity was recently presented in Reference [71] (see Figure 13). The tag was based on a M24LR04E-R from an ST NFC IC connected to a low-cost microcontroller (Atttiny85 from Atmel). The data are shown on a smartphone application or uploaded to the cloud for sharing or storage. The temperature is measured using an I^2^C temperature sensor (LM75A), while air humidity is detected by reading the analog output from the HIH-5030 humidity sensor from Honnewey, designed for measuring soil volumetric water content. Capacitance measurement is based on a low-power timer 555 working as an oscillator, and a diode detector whose output is measured by the ADC of the microcontroller. The external circuitry requires less than 1 mA at 3 V to operate. A procedure was presented for calibrating the sensor based on a simple expression whose coefficients can be experimentally obtained. Figure 14 shows a measurement taken with the system. This reference shows that conventional low-power sensors can be integrated within the NFC tag for the new generation of IoT devices.

## 4. Conclusions

We recently witnessed the rapid deployment of NFC technology driven by contactless payment applications. Although NFC technology was developed over a decade ago, it was not until its massive incorporation into mobile phones that it became popular. This expansion led to the emergence of passive NFC sensors using the energy-harvesting possibilities provided by this technology. In this paper, we reviewed recent studies found in the literature. We also addressed the design of labels based on energy harvesting, as well as several aspects that can limit the transfer of power between the tag and the reader. The inductance (and the corresponding capacitance) chosen in the tag design has an important role in the energy harvesting and the loading effects between the tag and reader. This interest in passive NFC sensors led manufacturers of integrated circuits to present several ICs with energy harvesting, thus demonstrating the potential market for this technology. We also reviewed some of these ICs and highlighted their main characteristics. A review of the state of the art in batteryless NFC sensors revealed great interest in these sensors for food monitoring and wearable biomedical applications. In these applications, it is essential to eliminate potentially dangerous batteries due to their toxicity and high costs. Compared with other types of wireless sensor technology, such as UHF RFID, an inductive link at the 13.56-MHz band is more insensitive, where the body introduces high losses that limit the read range. Another advantage of NFC devices over UHF devices is the fast ROI. This is because a specific reader it is not required, since a smartphone can often be used as a reader. The data can then be uploaded to cloud database services. The ease with which NFC technology is used makes it ideal for use by elderly people in telemedicine and electronic health applications. The greater privacy and security of NFC communications compared to UHF RFID is another point to consider in biomedical and telemedicine applications. If a specific sensor must be designed because one is not commercially available, integrating NFC electronics within the ASIC and the sensor signal conditioning is justified. In other cases, standard commercially available NFC ICs were used in the NFC-based designs found in the literature. These ICs are often based on the standard ISO 15693 because higher communication distances are obtained compared with the IC under the standard ISO 14443.

## Figures and Tables

**Table 1:** Comparison of radio-frequency identification (RFID) sensor technologies. NFC—near-field communication; UHF—ultra-high frequency; BLE—Bluetooth low energy; UWB—ultra-wideband; IC—integrated circuit; BAP—battery-assisted passive; ISM—industrial, scientific, and medical.

Property	Chipless RFID	NFC	UHF RFID	Bluetooth BLE
Typical read range	<50 cm frequency coded 2–3 m, time-coded UWB	1–2 cm for proximity cards with energy harvesting, 0.5 m for vicinity cards	Up to 15 m with inlay tags with −22 dBm read IC sensitivity. Up to 3 m UHF sensors (with −9 dBm read IC sensitivity). Up to 30 m BAP.	10 m
Power source	Passive	Passive or semi-passive	Passive or semi-passive	Active
Tag price	Moderate	Low	Low	High
Reader cost	High, no commercial	Low, smartphone	High, $1000–$2000	Low, smartphone
Standard	No	Yes	Yes	Yes
Universal frequency regulation	No, often used UWB	Yes, ISM	No, by regions	Yes, ISM
Tag size	Large	Medium	Medium	Small
Memory capacity	<40 bits	<64 kilobits	96bits EPC, typically 512 bits for users (<64 kbytes)	Several kilobytes depending on the microcontroller
ID rewritable	No	Yes	Yes	Yes
Energy harvesting	No	Approx. 10 mW	Few µW	No
Tag substrate	Low loss microwave substrates	Low cost or FR4	Low cost or FR4	FR4
Tag flexibility	Depends on the substrate	Depends on the substrate	Depends on the substrate	No
Tag robustness	High	Low (inlays)	Low (inlays)	Moderate

**Table 2:** Comparison of commercial NFC ICs with energy harvesting. ADC—analog-to-digital converter; SPI—serial peripheral interface; NA—not applicable; UART—universal asynchronous receiver-transmitter.

IC	Energy Harvesting Maximum Sink Current and Typical Voltage	ADC	Bus	Comments
M24LR-E-R ST25DV-I2C ST Micro-electronics	6 mA/3 V	No	I^2^C	4–64 kbit ISO15693
NT3H1101 NT3H1201 NXP	5 mA/2 V	No	I^2^C	8 kbit/16 kbit ISO 14443-3
NF4 EM Micro-electronics	5 mA/3.6 V	No	SPI	8 kbit/32 kbit/64 kbit ISO14443A
GT23SC6699-1/2 Giantec Semiconductor	NA/3.2 V	No	I^2^C	8 kbit/16 kbit ISO 14443-3
SIC4310 SIC4340 SIC4341 Silicon Craft	10 mA/3.3V	No Yes	UART	220 bytes EEPROM ISO 14443A
AS3953A AMS AG	5 mA/2 V	No	SPI	1 kbit ISO14443A-4
SL13 AMS AG	4 mA/3.4 V	Yes	SPI	8 kbit ISO 15693 Temperature sensor integrated
MLX90129 Melexis	5 mA/3 V	Yes	SPI	4 kbit ISO-15693
RF430FRL152H Texas Instruments	NA/3 V	Yes	I^2^C/SPI	ISO15693 MSP430 2 kB FRAM

**Table 3:** Dielectric layers used in the simulation of the arm at 37 °C.

Layer	Relative Permittivity	Conductivity (S/m)	Thickness (mm)
Skin	120	0.25	1.5
Fat	38	0.21	4
Muscle	152	0.74	25
Bone	11	0.03	25

**Table 4:** The equivalent circuit of the antenna and tuning capacitance as a function of the location.

Case	Inductance (µH)	Capacitance *C_p_* (pF)	Resonance Frequency (MHz)	Antenna Quality Factor	Tuning Capacitance *C_tuning_* (pF) ^1^
On air	2.62	1.64	76.3	153	25.9
On body	2.62	9.02	32.7	6	18.5
On body with ferrite foil	3.34	6.70	37.5	131	9.5
On body with a foam spacer of 1 mm	2.62	2.78	58.9	91	24.8
On body with a plastic spacer of 1 mm	2.62	3.64	51.5	38	23.9
On body with a plastic spacer of 2 mm	2.62	2.88	57.9	92	24.7

^1^ Assuming *C_IC_* = 25 pF.

**Table 5:** Comparison of NFC-based sensors in the literature. Y—yes; N—no; ASIC—application-specific integrated circuit; ISFET—ion-sensitive field-effect transistor; ECG—electrocardiogram; TEG—thermoelectric generator; SWCNT—single-walled carbon nanotube; MEMS—microelectromechanical system.

Reference	Application	IC	Passive	Comments
[29]	Cold-chain temperature	NFC-WISP (ISO14443)	Y	Optional E-ink display Discrete rectifier
[55]	Temperature	NFC-WSIP (ISO14443)	N	Measurement of newborn temperature in an incubator
[56]	Cold-chain temperature	MLX90129 (ISO15693)	Y	Critical temperature indicator based on melting point
[60]	Biopatch body temperature	RF430FRL152H (ISO15693)	Y	Can be used as data logger if the battery is used Temperature measurement using thermistors
[61]	Tattoo body temperature and light	RF430FRL152H (ISO15693)	Y	Low-cost tattoo-like stretchable
[62]	Sweat, NaCl concentration	MLX90129 (ISO15693)	Y	Measures the potential between two electrodes for NaCl concentration in the sweat
[63]	Biopatch pH	SL13 (ISO15693)	Y	Microfluidic electrodes
[64]	pH	SIC4310 (ISO14443)	Y	ASIC with a 3 × 3 array of ISFET
[65]	ECG	Custom ASIC (ISO14443)	Y	Powered from energy-harvesting TEG and supercapacitor.
[66]	Implantable glucose monitor	Custom ASIC (ISO14443)	N	Glucose concentration is encoded in the modulating frequency Semi-passive
[67]	Glucose monitor	Custom ASIC (ISO14443)	Y	micro-fluorimeter.
[68]	Status food monitoring Multigas sensor	SL13 (ISO15693)	Y	PIC16LF1703 microcontroller, Color detector S11059-02DT
[69]	Gas sensor	Modified inlay (ISO14443)	Y	Functionalized SWCNTs On/off detection based on a change in the resonance frequency
[70]	Tire pressure	Custom ASIC (ISO14443)	Y	MEMS capacitive sensor
[71]	Temperature, humidity, soil moisture	M24LR04E-R (ISO15693)	Y	Attiny85 microcontroller LM75A temperature sensor HIH-5030 humidity sensor

## References

[1] Finkenzeller K., Müller D. (2010). RFID Handbook: Fundamentals and Applications in Contactless Smart Cards, Radio Frequency Identification and Near-Field Communication.

[2] Near Field Communications Forum. http://nfc-forum.org.

[3] Coskun V., Ozdenizci B., Ok K. (2013). A survey on near field communication (NFC) technology. Wirel. Pers. Commun..

[4] Ozdenizci B., Coskun V., Ok K. (2015). NFC internal: An indoor navigation system. Sensors.

[5] Carré F., Caudeville J., Bonnard R., Bert V., Boucard P., Ramel M. (2017). Soil contamination and human health: A major challenge for global soil security. Global Soil Security. Progress in Soil Science.

[6] Yildiz F. (2009). Potential Ambient Energy-Harvesting Sources and Techniques. J. Technol. Stud..

[7] Kim S., Vyas R., Bito J., Niotaki K., Collado A., Georgiadis A., Tentzeris M.M. (2014). Ambient RF Energy-Harvesting Technologies for Self-Sustainable Standalone Wireless Sensor Platforms. Proc. IEEE.

[8] Introduction to the Power Class 0 Specification, Wireless Power Consortium, Version 1.2.3, February 2017. https://www.wirelesspowerconsortium.com/downloads/download-wireless-power-specification.html.

[9] Choi B., Nho J., Cha H., Ahn T., Choi S. (2004). Design and implementation of low-profile contactless battery charger using planar printed circuit board windings as energy transfer device. IEEE Trans. Ind. Electron..

[10] Musavi F., Edington M., Eberle W. Wireless power transfer: A survey of EV battery charging technologies. Proceedings of the IEEE Energy Conversion Congress and Exposition (ECCE).

[11] Vijayaraman B.S., Osyk B.A. (2006). An empirical study of RFID implementation in the warehousing industry. Int. J. Logist. Manag..

[12] Lazaro A., Girbau D., Salinas D. (2009). Radio link budgets for UHF RFID on multipath environments. IEEE Trans. Antennas Propag..

[13] Bjorninen T., Sydanheimo L., Ukkonen L., Rahmat-Samii Y. (2014). Advances in antenna designs for UHF RFID 546 tags mountable on conductive items. IEEE Antennas Propag. Mag..

[14] Amendola S., Milici S., Marrocco G. (2015). Performance of epidermal RFID dual-loop tag and on-skin retuning. IEEE Trans. Antennas Propag..

[15] Marrocco G. (2010). Pervasive electromagnetics: Sensing paradigms by passive RFID technology. IEEE Wirel. Commun..

[16] Babar A.A., Manzari S., Sydanheimo L., Elsherbeni A.Z., Ukkonen L. (2012). Passive UHF RFID Tag for Heat Sensing Applications. IEEE Trans. Antennas Propag..

[17] Fernández-Salmerón J., Rivadeneyra A., Martínez-Martí F., Capitán-Vallvey L.F., Palma A.J., Carvajal M.A. (2015). Passive UHF RFID tag with multiple sensing capabilities. Sensors.

[18] De Donno D., Catarinucci L., Tarricone L. (2014). RAMSES: RFID Augmented Module for Smart Environmental Sensing. IEEE Trans. Instrum. Meas..

[19] Lorenzo J., Girbau D., Lazaro A., Villarino R. (2011). Read range reduction in UHF RFID due to antenna detuning and gain penalty. Microw. Opt. Technol. Lett..

[20] Tedjini S., Karmakar N., Perret E., Vena A., Koswatta R., E-Azim R. (2013). Hold the Chips: Chipless Technology, an Alternative Technique for RFID. IEEE Microw. Mag..

[21] Lazaro A., Ramos A., Girbau D., Villarino R. (2011). Chipless UWB RFID Tag Detection Using Continuous Wavelet Transform. IEEE Antennas Wirel. Propag. Lett..

[22] Costa F., Genovesi S., Monorchio A. (2013). A chipless RFID based on multiresonant high-impedance surfaces. IEEE Trans. Microw. Theory Tech..

[23] Vena A., Perret E., Tedjini S. (2012). High-Capacity Chipless RFID Tag Insensitive to the Polarization. IEEE Trans. Antennas Propag..

[24] Issa K., Alshoudokhi Y.A., Ashraf M.A., AlShareef M.R., Behairy H.M., Alshebeili S., Fathallah H. (2018). A High-Density L-Shaped Backscattering Chipless Tag for RFID Bistatic Systems. Int. J. Antennas Propag..

[25] Ramos A., Girbau D., Lazaro A., Villarino R. (2015). Wireless concrete mixture composition sensor based on time-coded UWB RFID. IEEE Microw. Wirel. Compon. Lett..

[26] Girbau D., Ramos A., Lazaro A., Rima S., Villarino R. (2012). Passive wireless temperature sensor based on time-coded UWB chipless RFID tags. IEEE Trans. Microw. Theory Tech..

[27] Lazaro A., Villarino R., Costa F., Genovesi S., Gentile A., Buoncristiani L., Girbau D. (2018). Chipless Dielectric Constant Sensor for Structural Health Testing. IEEE Sens. J..

[28] Butler P. (2012). Harvesting Power in a Near Field Communications (NFC) Device. U.S. Patent.

[29] Zhao Y., Smith J.R., Sample A. NFC-WISP: A sensing and computationally enhanced near-field RFID platform. Proceedings of the IEEE International Conference on RFID (RFID).

[30] Wikner J.J., Zötterman J., Jalili A., Farnebo S. Aiming for the cloud—A study of implanted battery-free temperature sensors using NFC. Proceedings of the International Symposium on Integrated Circuits (ISIC).

[31] Paret D. (2016). Design Constraints for NFC Devices.

[32] Zargham M., Gulak P.G. (2012). Maximum Achievable Efficiency in Near-Field Coupled Power-Transfer Systems. IEEE Trans. Biomed. Circ. Syst..

[33] Gebhart M., Szoncso R., Muenzer M. Improving contactless technology by increase of transponder load modulation with serial capacitor. Proceedings of the 15th IEEE Mediterranean Electrotechnical Conference.

[34] Youbok L. (2003). Antenna Circuit Design for RFID Applications. Microchip Application Note AN710.

[35] Kim D.H., Park Y.J. (2016). Calculation of the inductance and AC resistance of planar rectangular coils. Electron. Lett..

[36] Hirayama H. (2012). Equivalent Circuit and Calculation of Its Parameters of Magnetic-Coupled-Resonant Wireless Power Transfer. Wireless Power Transfer–Principles and Engineering Explorations.

[37] Lee B., Kim B., Harackiewicz F.J., Mun B., Lee H. (2014). NFC Antenna Design for Low-Permeability Ferromagnetic Material. IEEE Antennas Wirel. Propag. Lett..

[38] Li W., Chung W., Hsiao F., Li T., Kao T., Huang M. Compact multi-layer handset phone 13.56 MHz NFC antenna design by novel laser-induced thin-film antenna (LITA) technologies. Proceedings of the International Symposium on Antennas and Propagation (ISAP).

[39] Lee B., Harackiewicz F.J. (2017). Design of a Simple Structured NFC Loop Antenna for Mobile Phone Applications. Prog. Electromagn. Res..

[40] Zhu J., Ban Y., Sim C., Wu G. (2017). NFC Antenna with Nonuniform Meandering Line and Partial Coverage Ferrite Sheet for Metal Cover Smartphone Applications. IEEE Trans. Antennas Propag..

[41] Xiao S., Xiao H., Shan L. A study of application of ferrite sheet of periodic structure to NFC antenna. Proceedings of the 3rd IEEE International Conference on Computer and Communications (ICCC).

[42] Boada M., Lazaro A., Villarino R., Gil E., Girbau D. Near-Field Soil Moisture Sensor with Energy Harvesting Capability. Proceedings of the 48th European Microwave Conference.

[43] Gebhart M., Bruckbauer J., Gossar M. Chip impedance characterization for contactless proximity personal cards. Proceedings of the 7th International Symposium on Communication Systems, Networks & Digital Signal Processing (CSNDSP 2010).

[44] Ishii M., Komiyama K. A Measurement Method for the Antenna Factor of Small Loop Antenna by Measuring the Input Impedance. Proceedings of the Conference on Precision Electromagnetic Measurements.

[45] Gebhart M., Birnstingl S., Bruckbauer J., Merlin E. Properties of a test bench to verify standard compliance of proximity transponders. Proceedings of the 6th International Symposium on Communication Systems, Networks and Digital Signal Processing.

[46] Qing X., Chen Z.N. (2007). Proximity Effects of Metallic Environments on High Frequency RFID Reader Antenna: Study and Applications. IEEE Trans. Antennas Propag..

[47] Zhu H., Lai S., Dai H. Solutions of Metal Surface Effect for HF RFID Systems. Proceedings of the International Conference on Wireless Communications, Networking and Mobile Computing.

[48] Gebhart M., Neubauer R., Stark M., Warnez D. Design of 13.56 MHz Smartcard Stickers with Ferrite for Payment and Authentication. Proceedings of the Third International Workshop on Near Field Communication.

[49] Pethig R. (1984). Dielectric properties of biological materials: Biophysical and medical applications. IEEE Trans. Electr. Insul..

[50] Huang J.C., Lin Y., Yu J.K., Liu K., Kuo Y. A Wearable NFC Wristband to Locate Dementia Patients through a Participatory Sensing System. Proceedings of the International Conference on Healthcare Informatics.

[51] Bindroo O., Saxena K., Khatri S.K. A wearable NFC wristband for remote home automation system. Proceedings of the 2nd International Conference on Telecommunication and Networks (TEL-NET).

[52] Kajfez D.P., Guillon P. (1998). Dielectric Resonators.

[53] Number of NFC-Enabled Mobile Devices Worldwide from 2012 to 2018 (In Million Units). https://www.statista.com/statistics/461494/nfc-enabled-mobile-devices-worldwide/.

[54] Consultancy: 400% Increase in NFC Shipments 2018. https://www.secureidnews.com/news-item/consultancy-400-increase-in-nfc-shipments-2018/#.

[55] de Oliveira J.I., do Prado Villarroel Zurita M.E. Development of NFC TAG for temperature sensing of premature newborns in neonatal incubators. Proceedings of the 2nd International Symposium on Instrumentation Systems, Circuits and Transducers (INSCIT).

[56] Lorite G.S., Selkälä T., Sipola T., Palenzuela J., Jubete E., Viñuales A., Cabañero G., Grande H.J., Tuominen J., Uusitalo S. (2017). Novel, smart and RFID assisted critical temperature indicator for supply chain monitoring. J. Food Eng..

[57] Strommer E., Kaartinen J., Parkka J., Ylisaukko-oja A., Korhonen I. (2006). Application of near field communication for health monitoring in daily life. Proceedings of the 28th Annual International Conference of the IEEE Engineering in Medicine and Biology Society.

[58] Jara A.J., Lopez P., Fernandez D., Zamora M.A., Ubeda B., Skarmeta A.F. (2013). Communication protocol for enabling continuous monitoring of elderly people through near field communications. Interact. Comput..

[59] Milici S., Lazaro A., Villarino R., Girbau D., Magnarosa M. (2018). Wireless Wearable Magnetometer-Based Sensor for Sleep Quality Monitoring. IEEE Sens. J..

[60] Vicente J.M., Avila-Navarro E., Juan C.G., García N., Sabater-Navarro J.M. Design of a wearable bio-patch for monitoring patient’s temperature. Proceedings of the 38th Annual International Conference of the IEEE Engineering in Medicine and Biology Society (EMBC).

[61] Jeong H., Ha T., Kuang I., Shen L., Dai Z., Sun N., Lu N. NFC-enabled, tattoo-like stretchable biosensor manufactured by “cut-and-paste” method. Proceedings of the 39th Annual International Conference of the IEEE Engineering in Medicine and Biology Society (EMBC).

[62] Rose D.P., Ratterman M., Griffin D.K., Hou L., Kelley-Loughnane N., Naik R.K., Hagen J.A., Papautsky I., Heikenfeld J. System-level design of a RFID sweat electrolyte sensor patch. Proceedings of the 36th Annual International Conference of the IEEE Engineering in Medicine and Biology Society.

[63] Rahimi R., Brener U., Ochoa M., Ziaie B. Flexible and transparent pH monitoring system with NFC communication for wound monitoring applications. Proceedings of the 30th International Conference on Micro Electro Mechanical Systems (MEMS).

[64] Douthwaite M., Georgiou P. Live demonstration: An NFC based batteryless CMOS ISFET array for real-time pH measurements of bio-fluids. Proceedings of the IEEE SENSORS.

[65] Wu C.C., Kuo W.C., Wang H.J., Huang Y.C., Chen Y.H., Chou Y.Y., Yu S.A., Lu S.S. A pliable and batteryless real-time ECG monitoring system-in-a-patch. Proceedings of the VLSI Design, Automation and Test (VLSI-DAT).

[66] Anabtawi N., Freeman S., Ferzli R. A fully implantable, NFC enabled, continuous interstitial glucose monitor. Proceedings of the IEEE-EMBS International Conference on Biomedical and Health Informatics (BHI).

[67] De Hennis A., Getzlaff S., Grice D., Mailand M. (2016). An NFC-Enabled CMOS IC for a Wireless Fully Implantable Glucose Sensor. IEEE J. Biomed. Health Inform..

[68] Escobedo P., Erenas M.M., Lopez-Ruiz N., Carvajal M.A., Gonzalez-Chocano S., de Orbe-Paya I., Capitan-Valley L.F., Palma A.J., Martinez-Olmos A. (2017). Flexible passive near field communication tag for multigas sensing. Anal. Chem..

[69] Azzarelli J.M., Mirica K.A., Ravnsbæk J.B., Swager T.M. (2014). Wireless gas detection with a smartphone via rf communication. Proc. Natl. Acad. Sci. USA.

[70] Kollegger C., Greiner P., Steffan C., Wiessflecker M., Froehlich H., Kautzsch T., Holweg G., Deutschmann B. A system-on-chip NFC bicycle tire pressure measurement system. Proceedings of the 60th IEEE International Midwest Symposium on Circuits and Systems (MWSCAS).

[71] Boada M., Lazaro A., Villarino R., Girbau D. (2018). Battery-Less Soil Moisture Measurement System Based on a NFC Device with Energy Harvesting Capability. IEEE Sens. J..

